# Central retinal artery and vein occlusion as a complication of persistent hyaloid artery – a case report

**DOI:** 10.1186/s12886-020-01702-8

**Published:** 2020-11-03

**Authors:** Mirjana Bjeloš, Ana Križanović, Mladen Bušić, Biljana Kuzmanović Elabjer

**Affiliations:** 1 Department of Ophthalmology, Reference Centre for Paediatric Ophthalmology and Strabismus of the Ministry of Health of the Republic of Croatia, Sveti Duh University Hospital, Sveti Duh 64, Zagreb, Croatia; 2 Faculty of Medicine, Josip Juraj Strossmayer University of Osijek, Osijek, Croatia; 3 Faculty of Dental Medicine and Health Osijek, Josip Juraj Strossmayer University of Osijek, Osijek, Croatia

**Keywords:** Persistent hyaloid artery, Torsion, Retinal artery occlusion, Retinal vein occlusion, Case report

## Abstract

**Background:**

In this case report, we present for the first time central retinal artery occlusion (CRAO) and central retinal vein occlusion (CRVO) as a complication of persistent hyaloid artery (PHA).

**Case presentation:**

In August 2019, a six-year-old male patient manifested right eye (RE) excessive tearing, conjunctival injection and pain. On examination, RE demonstrated light perception and intraocular pressure of 36 mmHg. The diagnoses of neovascular glaucoma, CRVO and CRAO were established as affirmed with fluorescein angiography (FA). PHA was not reported. Extensive work-up and family history were unremarkable. The child was born on term after uncomplicated twin pregnancy. In December 2019, he was referred to our Centre. Transillumination revealed fully dilated, non-reactive RE pupil, clear lens and tubular remnant of HA containing blood cells in its lumen freely rotating in the anterior vitreous.

**Conclusions:**

PHA results from failure of apoptosis during gestation. It can easily be observed during the red reflex screening at neonatal wards. We hypothesized that PHA twisting led to torsion of the residual primordial common bulb, branching off to HA and CRA with CRAO occurring first. The consequential CRVO presumably advanced by venous stasis due to decrease in arterial inflow. Liquid vitreous appears as early as 4 years of age enabling PHA to whirl more freely. Thus, in case of PHA, we advocate FA to be performed and if connection with retinal artery is proven, parents should be informed on the possible devastating complications and prompt surgical treatment should be considered.

**Supplementary Information:**

The online version contains supplementary material available at 10.1186/s12886-020-01702-8.

## Background

Persistent hyaloid artery (PHA) results from failure of apoptosis of the hyaloid vascular system [1]. It is characterized by abnormally firm attachment of the vitreous to the posterior pole that prevents complete posterior hyaloid separation contributing to epiretinal membrane formation and possible vitreoretinal traction [2].

Atrophy of the HA is usually complete before birth, but anatomical remnants of the hyaloid system may present as Mittendorf’s dot at the posterior lens capsule or Bergmeister’s papilla at the optic disc [2, 3]. PHA is seen in 3% of full term infants and 95% of premature infants [4]. However, PHA with active blood flow is a rare finding of unknown incidence [2, 3, 5], but when present may be supplying a part of the retinal tissue [3, 5, 6].

PHA is usually associated with mild amblyopia without further complications, although nystagmus, strabismus, cataract, vitreous haemorrhage and retinal detachment have been reported [5, 7]. Vitreous haemorrhage in cases of PHA can develop due to tractional force during rapid eye movement phase of sleep, external trauma to the globe, elevated blood pressure, as well as spontaneously [5]. We herein report for the first time consequential central retinal artery occlusion (CRAO) and central retinal vein occlusion (CRVO) as a complication of PHA in a six-year-old child.

## Case presentation

In August 2019, a six-year-old male patient manifested right eye (RE) excessive tearing, conjunctival injection and pain. Family history was unremarkable. The child was born after uncomplicated twin pregnancy, in 37th gestational week, but ultrasound assessment was consistent with 34th maturity week. Birth weight was 2100 g and Apgar score 9/10. Photographs before the event depicted isocoria without anterior segment asymmetry.

On examination, RE demonstrated visual acuity of light perception and intraocular pressure (IOP) of 36 mmHg. Left eye showed no abnormalities. The diagnoses of neovascular glaucoma, CRVO and CRAO were established as affirmed with fluorescein angiography (FA). Extensive systemic work-up was performed. Neurological and cardiologic examinations were unremarkable. Laboratory tests for tuberculosis, Epstein-Barr virus, cytomegalovirus, *Toxoplasma gondii*, rubella, herpes simplex virus type 1 and 2, and HIV were negative, as well as factor V Leiden (FVL) mutation, MTHFR gene, anticardiolipin antibodies, and lupus anticoagulant. PHA was not reported in any of the findings. The child’s condition was perceived as idiopathic CRAO and CRVO, which led to the development of neovascular glaucoma. The patient was treated with antiglaucoma drugs topically (dorzolamide + timolol 20 mg/L + 5 mg/L drops b.i.d., latanoprost 50 μg/mL drops q.d., and acetazolamide 250 mg q.d.), panretinal photocoagulation, ranibizumab intravitreally and cyclophotocoagulation.

**Fig. 1:**
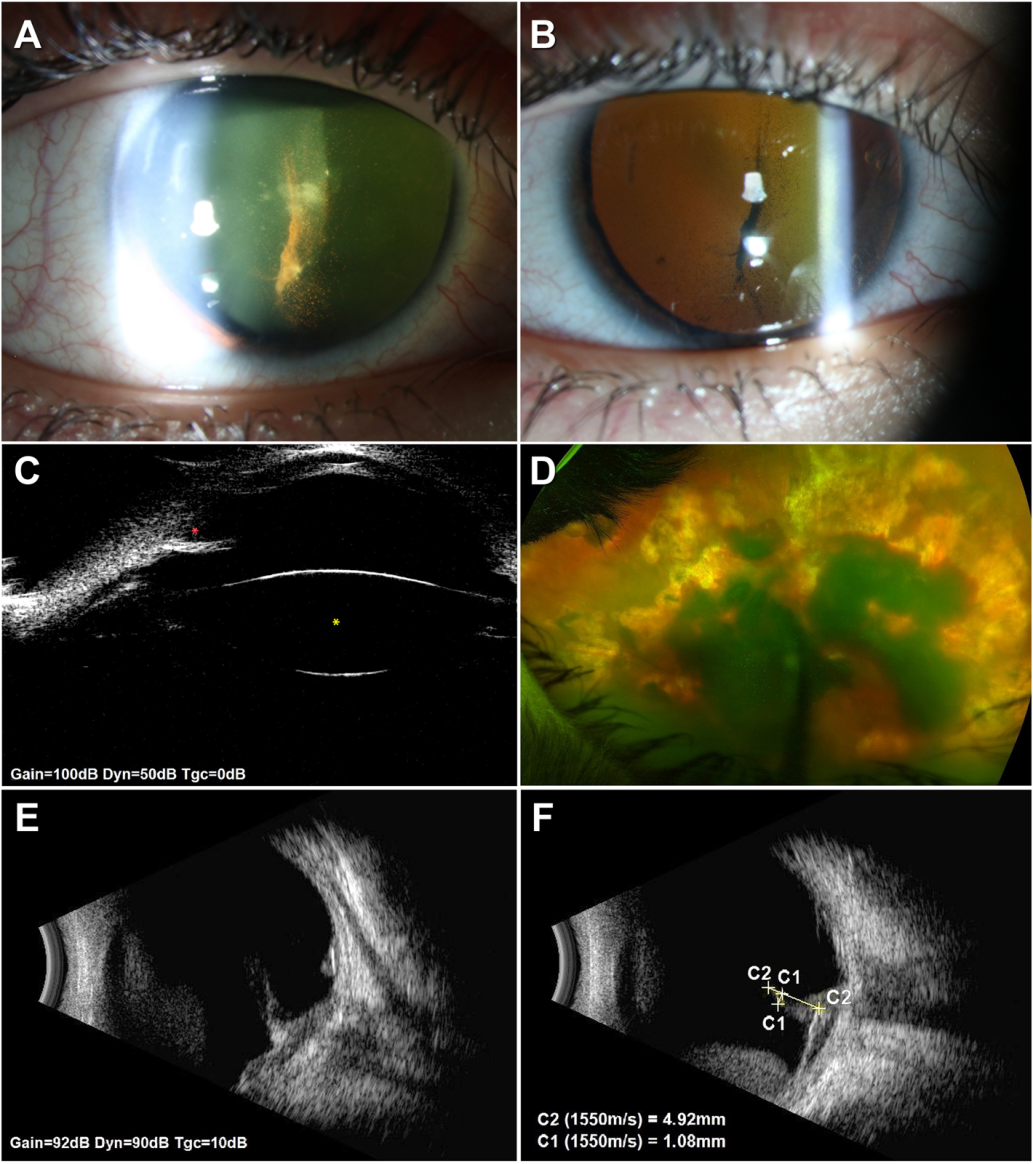
**a** The slit-lamp indirect illumination reveals dilated, non-reactive pupil and red blood cells in the anterior vitreous adjoining hyaloid artery remnant. **b** Retroillumination image. **c** Ultrasound biomicroscopy delineates closed angle (red asterisk) and clear lens (yellow asterisk). **d** Wide field fundus photograph. Massive subhyaloid and vitreous haemorrhages obscure the posterior pole and fundus periphery visualization. **e** Ultrasound B-scan images depict low reflective membranous tubular structure of the persistent hyaloid artery originating within the optic nerve head. It extends into the vitreous demonstrating substantial after-movements. **f** Markings indicate the size of the hyaloid artery, measuring 4.92 × 1.08 mm

In December 2019, the child was referred to our Centre for second opinion. Clinical examination showed no light perception. The slit-lamp indirect lateral illumination revealed fully dilated, non-reactive RE pupil (Fig. 1a). Retroillumination verified blood cells in the HA lumen and anterior vitreous (Fig. 1b). RE IOP was 38 mmHg. Brückner’s transillumination revealed dim red reflex and tubular remnant of HA freely rotating in the anterior vitreous (Additional file 1). Closed angle and clear lens were depicted using ultrasound biomicroscopy (Fig. 1c). Extensive subhyaloid and intravitreal haemorrhages obscured visualization of fundus periphery and posterior pole (Fig. 1d). Thus, RE optical coherence tomography (OCT) angiography could not be performed. Ultrasound B-scan affirmed low reflective membranous tubular structure of the persistent hyaloid artery extending freely into the vitreous demonstrating substantial after-movements (Fig. 1e). It originated within the optic nerve head measuring 4.92 × 1.08 mm (Fig. 1f).

Vascular occlusion incident was discerned as a consequence of the existence of patent HA in the highly mobile Cloquet’s canal. No therapeutic intervention was currently indicated, but only close follow-up because the patient did not complain of any eye pain.


**Additional file 1.** Video demonstrating tubular remnant of hyaloid artery rotating extensively in the anterior vitreous of the right eye.

## Discussion and conclusions

Of the major vascular systems in the human eye, the choriocapillaris and foetal vasculature of the vitreous, including the hyaloid vasculature, *vasa hyaloidea propria* and *tunica vasculosa lentis*, develop first by hemo-vasculogenesis around 4–6 gestational weeks [1]. This is followed by angiogenesis of intermediate choroidal blood vessels, budding from the choriocapillaris [1]. Vasculogenesis of the human retinal vasculature is the last to evolve [1].

The HA, a branch of the ophthalmic artery, is located in the optic canal and extends from the optic disc to the crystalline lens through the vitreous humour [3]. HA branches throughout the vitreous, forming the *vasa hyaloidea propria* [8]. This vascular network forms anastomoses with the *tunica vasculosa lentis*, the vasculature surrounding the lens [8]. The anterior foetal vasculature supplies the developing lens and inner retina with oxygen and nutrients until the retinal vasculature forms [1].

HA is formed by 7 weeks of gestation [1]. The vasculature expands and matures throughout 12 weeks of gestation, and, at this stage, proliferation ensues in both endothelial cells and pericytes [1]. This angiogenic growth is led by the vascular endothelial growth factor (VEGF) mainly secreted by astrocytes [9]. At 13 weeks of gestation, regression and apoptosis of hyaloid vasculature is triggered [1, 10], terminating around the middle of the eighth month of gestation with retinal vasculature almost fully developed [11]. The programmed endothelial cell apoptosis and regression of hyaloid network is facilitated by physical separation of the VEGF-producing cells, due to differentiation of the lens epithelial cells into lens fibre cells and formation of the lenticular capsule [12], macrophages that secrete factors such as Wnt7b [13] and neurons [14, 15]. By titrating VEGF in retina, neurons limit angiogenesis through up-regulation of VEGF receptor 2 (VEGFR2), sequestering excessive VEGF [14, 15]. The absence of neuronal VEGFR2 results in misdirected angiogenesis, impeding transition from the foetal to postnatal circulatory network [14, 15].

In a healthy foetus, regression of the HA itself starts at 18 weeks, and by 29 gestational weeks it disappears leaving clear central zone in the vitreous humour, called Cloquet’s canal [3, 5, 16]. The main trunk of the HA finally closes centrally by the end of gestation [11].

Therefore, based upon gestational age, the hyaloid system is expected to be persistent in many very low birth weight infants [17], as in case of our patient. However, at the time of birth, some remnants of HA may persist and exceptionally the entire HA remains, constituting PHA [7].

There is a single case reporting combined CRVO and CRAO in a patient with PHA caused by cataract surgery [7]. The authors hypothesized that during hydrodissection, sudden disruption of the joining tissue caused arterial wall dissection and CRA collapse [7]. Consequently, the formation of this compartment could have generated a spatial compromise, giving rise to secondary CRVO [7]. Combined CRAO and CRVO in patients with FVL mutation, even though very rare, is described in the literature [18]. However, genetic analysis excluded this cause in our patient.

Contrary to the secondary cause [7], this case presented spontaneous combined CRAO and CRVO event related to patent PHA. We speculated that in our patient, PHA twisting led to torsion of the residual primordial common bulb, branching off to HA and CRA. In this case, we considered that CRAO would have occurred first, opposed to the inflammatory and thrombotic aetiology of combined CRVO and CRAO [18, 19]. The consequential CRVO advanced by venous stasis due to decrease in arterial inflow. As torsion itself caused irreversible ischemic injury, vision loss and neovascular glaucoma resulted.

By the third year of age, the vitreous is primarily a collagen gel. Thereafter, liquefaction occurs typically in its central part, suggesting that there is a gradual change in the vitreous collagen [7]. More liquid vitreous enables PHA to whirl more freely. The limitations of this case report include limited and insufficient quality of the clinical data prior to our examination. We cannot entirely exclude external factors such as physical activity or minor physical force applied to the eye as part of the gaming or other physical activity by the child, that may have led to the complication of PHA, which may have been unnoticed by the parents.

Thus, in case of PHA, we advocate FA to be performed and if connection with retinal artery is proven, parents should be informed on the possible devastating complications and vision loss. Accordingly, prompt surgical treatment should be considered.

## Data Availability

The data generated during the present study are available upon request from the corresponding author.

## Abbreviations

CRAO: Central retinal artery occlusion
CRVO: Central retinal vein occlusion
FA: Fluorescein angiography
FVL: Factor V Leiden
IOP: Intraocular pressure
PHA: Persistent hyaloid artery
RE: Right eye
VEGF: Vascular endothelial growth factor
VEGFR2: Vascular endothelial growth factor receptor 2
